# Mucormycosis and COVID-19-Associated Mucormycosis: Insights of a Deadly but Neglected Mycosis

**DOI:** 10.3390/jof8050445

**Published:** 2022-04-25

**Authors:** Laura C. García-Carnero, Héctor M. Mora-Montes

**Affiliations:** Departamento de Biología, División de Ciencias Naturales y Exactas, Campus Guanajuato, Universidad de Guanajuato, Noria Alta s/n, col. Noria Alta, C.P., Guanajuato 36050, Mexico

**Keywords:** COVID-19, SARS-CoV-2, mucormycosis, CAM, immunosuppression, hyperglycemia, corticosteroids, iron overload, ACE2, cytokine storm, GRP-78

## Abstract

The ongoing COVID-19 pandemic has quickly become a health threat worldwide, with high mortality and morbidity among patients with comorbidities. This viral infection promotes the perfect setting in patients for the development of opportunistic infections, such as those caused by fungi. Mucormycosis, a rare but deadly fungal infection, has recently increased its incidence, especially in endemic areas, since the onset of the pandemic. COVID-19-associated mucormycosis is an important complication of the pandemic because it is a mycosis hard to diagnose and treat, causing concern among COVID-19-infected patients and even in the already recovered population. The risk factors for the development of mucormycosis in these patients are related to the damage caused by the SARS-CoV-2 itself, the patient’s overstimulated immune response, and the therapy used to treat COVID-19, causing alterations such as hyperglycemia, acidosis, endothelial and lung damage, and immunosuppression. In this review, the molecular aspects of mucormycosis and the main risk factors for the development of COVID-19-associated mucormycosis are explained to understand this virus–fungi–host interaction and highlight the importance of this neglected mycosis.

## 1. Introduction

In late 2019, the novel severe acute respiratory syndrome coronavirus 2 (SARS-CoV-2), the etiological agent of the coronavirus disease 2019 (COVID-19), was firstly associated with human disease [1,2]. SARS-CoV-2 was identified in China on 12 December 2019, and since then, it has become an important health threat worldwide, being declared by the World Health Organization on 11 March 2020 as the etiological agent of the ongoing pandemic [3].

Like in other viral diseases, secondary infections are important COVID-19 complications, which might be caused by other viruses, bacteria, protozoa, and fungi [4]. At the beginning of the pandemic, it was reported that about 1% of the secondary infections in COVID-19 patients were caused by fungal pathogens [5,6,7], and several factors are thought to contribute to this. The patient’s preexisting health conditions, such as diabetes, immunosuppressive and antimicrobials agents misuse, and a long stay in the hospital may contribute to the development of secondary infections [8].

COVID-19-associated mucormycosis (CAM) is a severe emerging systemic fungal infection caused by several species of the *Mucorales* order [9,10], and it is already considered an epidemic in India [5,9,11,12]. However, CAM cases have also been reported worldwide, including in Brazil, Chile, Honduras, Paraguay, Uruguay, Mexico, the United States, the United Kingdom, Italy, France, Austria, and Iran [12,13,14,15,16,17,18,19,20,21,22,23,24,25,26,27,28]. Thus, it is a global health threat.

In this review, we will explain the molecular aspects of mucormycosis and discuss CAM’s main physiopathological characteristics and the fundamentals of its development to understand this pathogen–host interaction.

## 2. Mucormycosis

Mucormycosis, previously known as zygomycosis, is a rare opportunistic, invasive, and deadly fungal infection that has increased in incidence in the last few years, as a secondary infection in patients with debilitating diseases, such as diabetes, cancer, and organ transplantation, and during natural disasters, such as tsunamis and earthquakes [29]. Despite the availability of treatment options for mucormycosis, mortality rates range between 50% and 100% [30], which highlights the importance of this emerging disease.

Although precise data about the prevalence of this mycosis are unknown, due to the lack of diagnosis, according to epidemiological data of autopsy reports on invasive fungal diseases from 2008 to 2013, the etiological agents of mucormycosis were the fourth most common cause of invasive mycoses in the general population and the third most common cause in the onco-hematological and stem cell transplant populations, with a high prevalence of severe cases (around 70%) [31]. This infection is widely found in developing countries, being highly reported in India with uncontrolled diabetes as the main cause, while in developed countries, it is mainly found in patients with hematologic malignancies and hematopoietic stem cell transplantation [32,33].

The transmission route for these fungi can be through spores inhalation, contaminated food ingestion, traumatic inoculation with contaminated materials, or implantation in already abraded skin [34,35], which results in the development of several forms of the disease, including rhino-cerebral/rhino-orbital, pulmonary, gastrointestinal, cutaneous, and other disseminated forms. The disseminated disease is caused by the fungal angioinvasive capacity [36], with the rhino-cerebral form being the most common one in developing countries [32]. Regardless of the clinical form, patients show hemorrhage, thrombosis, infarction, and tissue necrosis [37], and the mortality rate depends on the organ affected, the causative agent, the patient’s health condition, and early diagnosis and treatment [38].

The mucormycosis causative agents are fast-growing thermotolerant fungi belonging to the *Mucorales* order, under the subphylum *Mucormycotina* and the phylum *Glomeromycota* [39,40]. *Rhizopus* is the genus most frequently associated with the disease, followed by *Mucor* and *Lichtheimia*, while *Zygomycetes* genera, including *Saksenae*, *Cunninghamella*, *Apophysomyces*, and *Rhizomucor*, are less common [35,40,41]. These organisms form a heterogenous group and are saprobes or facultative parasites, found worldwide growing on decaying organic matter, agricultural and forest soils, and animal feces, all with similar morphologies characterized by large aseptate/pauci-septated, ribbon-like hyaline hyphae with irregular or right-angle branching [36,38,42]. Most of these species are heterothallic [43,44] and reproduce asexually forming nonmotile mitospores of 3–11µm in diameter, produced in multi- or few-spored sporocarps, but there are a few species reported as homothallic, which are self-fertile [34,36].

Although all *Mucorales* share many unique characteristics, there are important differences among species in their epidemiology, pathogenesis, virulence, susceptibilities to the host immune response and antifungals, disease severity, and outcome [45,46,47]. However, most of the research has focused on *Rhizopus oryzae*, which is the most common mucormycosis causative agent [36].

### 2.1. Risk Factors

As mentioned before, these fungi are ubiquitously found in the environment, and their ability to cause disease in immunocompetent hosts is anecdotic but possible [48]. In the majority of the cases, the patients have underlying health conditions [40,49,50]. These patients have comorbidities or certain conditions that cause impairment of the host immune defenses and immunosuppression directly or indirectly [29,49], such as:

- Conditions that cause decreased numbers of phagocytes and altered immune functions, including hematologic malignancies, hematopoietic stem cell transplantation, solid organ malignancies and transplantation, and rheumatological diseases [10,40,42,49];
- Uncontrolled diabetes, diabetic ketoacidosis, and metabolic acidosis, which are conditions that cause changes in pH and iron availability within the host [10,29,40,42,49,51];
- The use of corticosteroids/immunosuppressors in high doses and for long periods [40,42], chelator therapy with deferoxamine (DFO) [10,29,40,42,49], and prolonged use of voriconazole [40,42,52];
- Malnutrition and neonatal prematurity [42,49]. On the other hand, the main factors linked to the development of mucormycosis in immunocompetent patients are:
- Traffic and home accidents, trauma, skin injuries, and burns [10,29,32,33,41];
- Natural disasters, such as tsunamis, tornadoes, and volcanic eruptions [29,34,41,53];
- The use of contaminated medical materials, such as bandages and tongue depressors, and prolonged hospital stays [32,34].

### 2.2. Pathogenesis

*Mucorales* have many traits that contribute to their ability to cause such an aggressive disease, including thermotolerance [54,55], rapid growth and angioinvasive nature [54,56], cell wall remodeling to endure hostile environments [56], iron uptake from the host [34,36,57], ability to bind to glucose-regulated proteins on endothelial cells [58], downregulation of host genes involved in the immune response and tissue repair [50,59], and resistance to most available antifungals [60].

The fungus might enter the host through different vias, including spore inhalation, skin inoculation, or ingestion through the gastrointestinal tract [50]. Independently of the inoculation route, the establishment of the infection depends on several steps: inoculation of spores, immune response evasion, attachment to the endothelium, endocytosis, germination into hyphae, endothelial damage, and hematogenous dissemination [41,50,55]. Thus far, the most studied *Mucorales* virulence factors that participate in the infection process are the attachment to endothelial cells and iron uptake. *Mucorales* are vasotropic; therefore, the interaction between the fungus and endothelial cells around blood vessels is an indispensable step in mucormycosis pathogenesis [29,40], which explains the angioinvasive nature of these organisms. 

Once in the host, spores attach to the basement membrane extracellular matrix proteins, laminin and collagen IV, and secrete proteolytic enzymes [61,62,63], subtilases [64], and lipolytic/glycosidic enzymes [65], which contribute to the destruction of the host tissue, which could have been already damaged by other host conditions, such as hyperglycemia or chemotherapy [36]. This interaction with the endothelial cells is specific and is through the spore-coat homolog (CotH) proteins [66], which bind to the receptor glucose-regulator protein 78 (GRP-78) [58]. It is well-reported that the expression levels of the CotH proteins determine the fungal virulence degree since high-virulent genera, such as *Rhizopus*, *Mucor*, and *Lichtheimia*, have three to seven CotH copies expressed, while low-virulent genera, such as *Apophysomyces*, *Cunninghamella*, *Saksenaea*, and *Syncephalastrum,* express only one to two copies, and *Entomophthorales* isolates, previously considered close to *Mucorales*, have no CotH genes and are avirulent [67].

The GRP-78 recognition by CotH proteins causes host cell death by the induction of endothelial cell-mediating endocytosis, initiating the fungal invasion of the endothelium [67]. The expression of both CotH and GRP-78 is highly enhanced in a hyperglycemic environment and with high concentrations of iron in acid pH, which is the reason diabetes and ketoacidosis are important risk factors for the development of mucormycosis [36,58]. GRP-78 binding to CotH proteins has been recognized as a unique mechanism for the *Mucorales* order since other opportunistic pathogens, such as *Candida albicans* and *Aspergillus fumigatus*, do not bind this endothelial cell receptor [58]. However, it is not the only factor involved in *Mucorales* binding to the host’s cells since blockage or suppression of GRP-78 does not completely abolish endothelial invasion [29]. In this regard, the platelet-derived growth factor receptor B (PDGFRB), found in the transcriptome of endothelial cells interacting with *Rhizopus delemar*, *R. oryzae*, and *Mucor circinelliodes* [67], also contributes to the fungus growth and angioinvasion since inhibition of PDGFRB phosphorylation partially reduces *Mucorales*-mediated endothelial damage in vitro [67].

When talking about fungal growth and replication, nutrition is a very important factor, and iron is essential for these processes. In *Mucorales*, iron uptake is performed by three mechanisms: low molecular weight iron chelators or siderophores, high-affinity iron permeases, and heme-oxygenases [34,36]. In healthy individuals, iron is bound to the serum proteins, but in diabetic patients with uncontrolled high glucose levels and ketoacidosis, iron is found unbound and in elevated concentrations, improving *R. oryzae* growth and causing phagocytosis defects [68,69]. In hyperglycemia conditions, excessive iron sequestering proteins glycation, such as transferrin, ferritin, and lactoferrin, is induced, which reduces iron affinity and increases free iron levels [29] while increasing CotH and GRP-78 expression [58,70]. Under acidic conditions due to the accumulation of ketone bodies, such as β-hydroxy butyrate, the transferrin capacity of chelating iron is impaired [51], and the GRP-78 expression is also increased [70]. Mucorales have an intrinsic siderophore known as rhizoferrin, which supplies the fungus with unbound iron through a receptor-mediated process [71,72] that, however, is incapable of obtaining iron bound to serum proteins [72,73], which is thought to be the main mechanism for iron uptake under hyperglycemic and acidic conditions. However, for iron uptake, *Rhizopus* is capable of using external siderophores, such as DFO, an iron chelator used as a treatment in patients with increased iron overload [36,73]. DFO chelates iron from transferrin and forms the complex iron-DFO, known as ferroxamine, which binds to the fungal cell surface receptors Fob1 and Fob2, where the iron from the complex is reduced [72,73,74], reoxidized, and transported intracellularly by the high-affinity iron permease FTR1 [57,72]. The use of DFO plays an important predisposing role in the development of highly lethal and disseminated mucormycosis [73]. FTR1 permease has been proved to be important for fungal pathogenicity since during infection, *R. oryzae* expresses *FTR1*, which, when inhibited or reduced, causes fungal virulence reduction [57]. 

Finally, hemoglobin can also be used as a source of ferric iron by *Mucorales*, to which they have access due to their angioinvasive ability [36]. Once the fungus transports hemoglobin intracellularly, the heme-oxygenases in the cytoplasm, through the reductase-permease system, enable the iron uptake from the host hemoglobin [56,57,72]. It is thought that during this process, FTR1 acts as a cytoplasmic membrane permease that facilitates intracellular heme uptake [34].

### 2.3. Immune Response during Mucormycosis

The main host defense against the *Mucorales* spores germination and dissemination are innate immune cells, consisting of circulating neutrophils, mononuclear cells, and macrophages [75]. Tissue and alveolar macrophages phagocytose the spores and kill them, and in the case where any spore survives and germinates, hyphae then induce neutrophils chemotaxis, which eliminate them through reactive oxygen metabolites, cationic peptides, perforin, and the production of proinflammatory cytokines, such as TNFα, INFγ, and IL-1β, which recruit and activate other immune cells [34,75,76,77]. However, other reports suggest that, although capable of phagocytosing *R. oryzae* spores and inhibiting their germination, macrophages cannot kill them due to the inhibition of LC3-associated phagocytosis (LAP), an important antifungal pathway that participates in the host defense regulation [78]. It was observed that melanin in the *Rhizopus* spore cell wall blocks phagosome responses, completely inhibiting phagosome maturation and allowing fungal intracellular persistence [75]. This fungistatic activity of macrophages and other phagocytes is thought to promote *Mucorales* virulence, prolonging fungal survival in the lungs and serving as transport of spores to other organs [78,79]. Therefore, it is suggested that macrophages’ fungicidal activity is dependent on the spores development stage since lack of cell wall remodeling and germination, which results in the retention of the cell wall melanin, inhibits LAP and therefore causes intracellular persistence [78,80]. In addition, recognition and phagocyte binding of *Mucorales* seem to be dependent on the spore wall composition [78], and the receptors participating in this process include Toll-like receptors, especially TLR-2, which plays an important role in *Rhizopus* recognition by neutrophils [75,76] and activation of proinflammatory cytokines such as IL-1β and TNFα [76].

Platelets also participate in the host immune response during this infection, as they strongly bind to *Mucorales* spores and hyphae. Once the platelets bind the pathogen and are activated, they damage hyphae in a time-dependent manner through the secretion of granules with pro- and anti-inflammatory cytokines and chemokines with antifungal properties [77,81] as well as increasing their tendency to aggregate, which enhances clot formation and suppresses hyphal growth and dissemination [47,77,81]. It was even observed that platelet antifungal activity is greater than that of polymorphonuclear leucocytes [81,82]. Additionally, these cells express membrane-bound molecules that bind to endothelial cells, monocytes, and dendritic cells, activating them and enhancing the immune response against the fungus [83].

Natural killer (NK) cells are also considered to have an important role in the immune response against *Mucorales*. It has been observed that NK cells are activated by *R. oryzae* hyphae and damage this morphology, mainly by the protein perforin [84,85] and depending on the amount of fungal biomass [86]. However, direct contact of *R. oryzae* hyphae with these cells decreases secretion of IFNγ and RANTES (Regulated upon Activation, Normal T-Cell Expressed, and Presumably Secreted), which are important molecules that improve the fungicidal activity of macrophages and the host defense against fungi [84,86]. Therefore, these cells’ activity is more effective in the early stage of the infection [85,86].

It has been demonstrated that *Mucorales* are capable of down-regulating genes involved in pathogen recognition, innate immune responses, and tissue repair mechanisms, facilitating fungal growth [87].

Although the knowledge regarding the adaptative response against *Mucorales* is scarce, it is already known that greater resistance to pulmonary mucormycosis is associated with an early Th-1 response, mediated by IFNγ and IL-2, while the infection control is mediated by the Th-17 response, with increased production of IL-17 and IL-2 by the spleen [88]. Just like in the pulmonary infection, in disseminated mucormycosis, IL-17 signaling and the Th1- response, through IFNγ signaling, are crucial for fungal clearance and thus control of the infection [88].

Finally, the patient’s health conditions, such as diabetes and acidosis, alter the immune response against *Mucorales*. It has been widely reported that hyperglycemia causes phagocyte dysfunction [89] and suppresses T lymphocyte induction and IFNγ production [36], while ketoacidosis impairs chemotaxis and neutrophils functions [34,59,65], all of them factors that favor the fungus growth and dissemination.

### 2.4. Treatment

Proper mucormycosis treatment depends on the etiological agent since the different *Mucorales* species have different drug resistance profiles. The main antifungals used for the treatment of this mycosis are amphotericin B, posaconazole, isavuconazole, and itraconazole, and they have different responses against the major agents of human infection, including *Lichtheimia* spp., *Rhizopus* spp., *Cunninghamella* spp., and *Mucor* spp. [90]. The most active antifungal for all *Mucorales* species is amphotericin B, with low minimum inhibitory concentrations (MICs) in vitro; however, higher MICs have been reported for this drug against *Cunninghamella* spp. and *Rhizopus* spp. [91,92,93,94,95,96]. On the other hand, posaconazole, isavuconazole, and itraconazole activity in vitro has been reported to be limited against *Mucorales* and highly dependent on the species, with higher MICs in vitro than amphotericin B; however, in vivo studies have demonstrated that azoles can be active against these pathogens: itraconazole can be effective against *Rhizomucor*, *Syncephalastrum*, and *Absidia*, but its effect is limited against *Cunninghamella* spp. and *Mucor* spp.; posaconazole has good effectivity against *Absidia* spp. and *Mycocladus corymbifera* but needs higher MICs for *Rhizopus* spp. and *Cokeromyces recurvatus*; and isavuconazole has partial in vitro activity against most *Mucorales* except for *Mucor circinelloides* [90,91,92,93,94,95,96,97,98]. In the case of voriconazole, it has been found to have a poor antifungal effect in vitro against most *Mucorales*, such as *Mucor* spp., *Rhizopus* spp., *Absidia* spp., *Cunninghamella* spp., and *Syncephalastrum* spp. [91]. Echinocandins have been reported to be active only against some *Mucorales* species in vitro but are thought to be a good option when used in combination with other drugs [93].

Combined therapy, such as antifungals plus surgical intervention, is usually related to a better mucormycosis outcome [99,100,101]. However, treatment preferences may vary depending on the mucormycosis clinical type: pulmonary, rhino-orbital/cerebral, and renal mucormycosis are treated preferably with concurrent antifungal and surgical therapy, while cutaneous mucormycosis can be treated just with antifungal drugs [102]. In most cases, if surgery and antifungal combined treatment are not administered on time, the disease progresses rapidly, causing the patient’s death [103].

Liposomal amphotericin B is the first-line antifungal monotherapy for the treatment of mucormycosis with the involvement of various organs [104], but different effects have been observed depending on the etiological agent genus and species [100]. Amphotericin B deoxycholate can also be used, but due to its toxicity in the doses and durations needed to treat mucormycosis [105,106], it is restricted to cases where no other antifungal therapy is available [104]. Surgical debridement and intravenous amphotericin B, along with the control of the patient’s underlying diseases, have proven to be an effective therapy for the control and treatment of mucormycosis [101].

Azoles such as posaconazole and isavuconazole are also considered first-line treatments [104]. Isavuconazole efficacy has been observed to be similar to amphotericin B [107,108] and is considered to be less hepatotoxic than other antifungals [109,110]. Posaconazole is also highly effective with a good tolerance [33,111], but due to its high oral bioavailability, the use of delayed-release tablets and intravenous formulation is recommended [112,113,114,115].

Evidence in animal models suggests that antifungal combination therapy can improve disease outcomes and survival rates [104]. Posaconazole in combination with amphotericin B or echinocandins shows a synergistic effect in vitro against some *Mucorales* species, a combination that might be used as good salvage therapy [116,117,118,119]. Moreover, posaconazole and amphotericin B in combination with surgical removal of damaged tissue have shown to be effective for aggressive mucormycosis infections [99,120,121]. In mucormycosis cases caused by trauma, combined therapy is especially recommended since mixed infection by several *Mucorales* species can be observed. For this, a combination of liposomal amphotericin B and either posaconazole or voriconazole can be used [122,123]. The reported cons of combined therapy are the potential added toxicity, drug interactions, and cost [104].

In the cases of refractory mucormycosis or toxicity to first-line therapy, some salvage treatments can be used. Itraconazole alone has been used to treat cutaneous mucormycosis caused by *M. irregularis* in patients with no underlying diseases and intolerance to amphotericin B [102] and, in combination with surgical treatment, leads to a favorable outcome in cases of cutaneous diseases in immunocompetent patients [118]. 

As already mentioned, the use of iron chelators such as deferoxamine is associated with a higher risk to develop this mycosis and a worsening of the disease, but newer iron chelators, such as deferiprone and deferasirox, have been successfully used to treat experimental mucormycosis [124].

In addition, immunotherapy strategies have also been used for the control of mucormycosis since anti-*Rhizopus* T cells from healthy donors show reactivity against *Mucorales* and enhance the phagocyte killing effect [125,126]. In addition, the use of IFNγ and GM-CDF stimulates human polymorphonuclear cells and increases *Rhizopus* hyphal damage [127]. A combination of immunotherapy and IFNγ was proven to be an effective treatment for intractable invasive mucormycosis [128].

## 3. COVID-19-Associated Mucormycosis

Although the first report of mucormycosis in the literature dates from 1855 [129], and despite having extremely high mortality rates [30], this infection has not received enough attention, as much information about the disease and its etiological agents is still unknown. However, with the surge of the COVID-19 pandemic, a rise in mucormycosis cases has been reported [12,130], highlighting the severity and importance of both diseases, especially when combined. Before the COVID-19 pandemic, mucormycosis had a mortality rate of 50%, but now, the mortality of CAM has increased to 85% in India not only due to the COVID-19 infection but also due to crowed hospitals and inadequate infrastructure, ineffective healthcare resources, lack of healthcare workers, poor diagnosis, and lack of awareness [131,132]. Currently, CAM represents 0.3% of fungal COVID-19 coinfections [133].

The increase in CAM development has been mainly observed during the second wave of the COVID-19 pandemic, which might suggest a more effective association between mucormycosis and the SARS-CoV-2 delta variant, probably because it is more contagious and resistant to vaccines, with a higher risk of hospitalization and predisposition for the rhino-cerebral form, and also because of this variant’s ability to affect the pancreas, predisposing to hyperglycemia [134].

The majority of CAM cases reported worldwide are from India, followed by the United States, Egypt, Iran, Brazil, and Chile, and a few cases have also been reported in the United Kingdom, France, Italy, Austria, and Mexico [135]. 

According to epidemiological reports, it has been observed that, just like COVID-19, CAM is mainly present in male patients with diabetes, hypertension, or treated with glucocorticoids [5,9,12,135,136,137,138,139,140,141]. Furthermore, in concordance with what has been observed in mucormycosis, the main clinical form of CAM is the rhino-orbital/cerebral, followed by pulmonary, cutaneous, disseminated, and gastrointestinal forms, with the most common etiological agents being *Rhizopus* spp. [12,130,135,141].

CAM has been reported in patients with active SARS-CoV-2 infection but also in patients already recovered, and although the majority of CAM cases were reported in severe COVID-19 patients, the mycosis has also been found in mild/moderate SARS-CoV-2 infections [135]. The first evidence of CAM is usually found 15 days after COVID-19 diagnosis, but it can take up to 90 days to detect the earliest symptoms [15,142]. 

**Figure 1:**
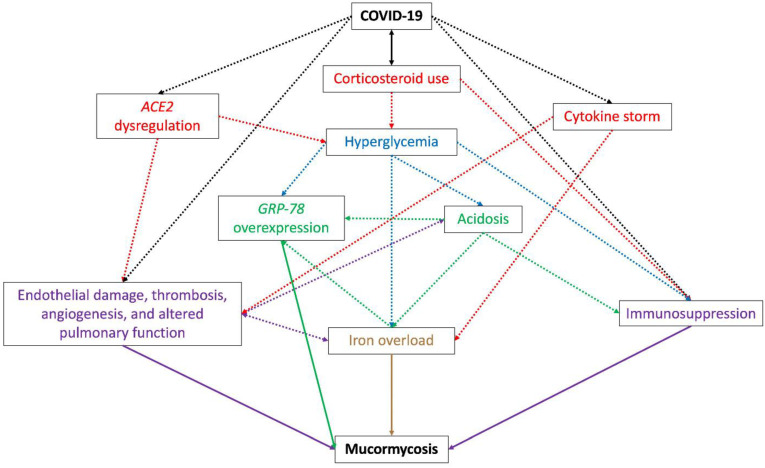
Schematic diagram representing the environment promoted by COVID-19 that predisposes the development of mucormycosis. The use of corticosteroids for COVID treatment and the dysregulation of ACE2 caused by SARS-CoV-2 create a hyperglycemic status in the patient, which causes overexpression of GRP-78, acidosis, and immunosuppression, this last also promoted by corticosteroid use, acidosis, and the COVID-19 itself. *GRP-78* overexpression and acidosis are closely related since the presence of one promotes the development of the other, both causing iron overload; this last is also promoted by the cytokine storm observed during COVID-19. ACE2 dysregulation is related to the development of endothelial damage, thrombosis, angiogenesis, and altered pulmonary function, which can also be caused by the SARS-CoV-2 replication and the cytokine storm. Iron overload, *GRP-78* overexpression, immunosuppression, endothelial damage, thrombosis, angiogenesis, and altered pulmonary function are directly related to mucormycosis development during COVID-19. Double black arrow: a risk factor for COVID-19 and mucormycosis infection. Dotted black lines: alterations directly caused by SARS-CoV-2 and COVID-19. Dotted red, blue, green, and purple lines: alterations caused by COVID-19. Purple, green, and brown lines: factors directly related to the development of mucormycosis.

To cause such an aggressive and lethal infection, the causative mucormycosis fungi take advantage of the many alterations that SARS-CoV-2 infection generates on the host, such as (Figure 1):

- Hyperglycemia, caused by the use of corticosteroids to treat COVID-19 [143] and dysregulation of the host receptor ACE2 (angiotensin-converting enzyme 2) observed during the viral infection [144,145] (explained in vii). Pre-existing diabetes is the main risk factor in most CAM cases [12] and is also related to an increase in the severity of SARS-CoV-2 infection [146];
- Low pH, caused by diabetic ketoacidosis or metabolic acidosis caused by corticosteroid use [147], which reduces the leukocytes’ phagocytic activity and impairs bronchoalveolar macrophages migration and functions [12,148];
- Immunosuppression, caused by SARS-CoV-2 infection and hypoxia, corticosteroids, and tocilizumab use [148,149] or patients’ previous comorbidities. Endothelial damage, thrombosis, and lymphopenia are usually observed during COVID-19, which contribute to the host immunosuppression [150,151];
- Free iron availability, caused by hyperglycemia, the COVID-19 cytokine storm, or by acidosis. Hyperglycemia and diabetic ketoacidosis cause dissociation of iron from ferritin and lactoferrin, thus elevating free iron concentrations [152], and some cytokines, mainly IL-6, stimulate ferritin synthesis and decrease iron export, increasing intracellular iron storage [153,154,155] and causing tissue damage and thus the release of iron into circulation [156]. Therapeutic intervention with lactoferrin has been suggested to revert iron availability [157,158,159];
- Pulmonary changes, including vascular endothielitis, thrombosis, and angiogenesis, caused by SARS-CoV-2 infection, which provides a focal point for fungal growth and dissemination [103,160]. The damage to alveolar cells can also promote anaerobic glycolysis, which causes lactic acidosis [145];
- Overexpression of endothelial cells GRP-78, caused by hyperglycemia, acidosis, and iron availability, which enables angioinvasion, hematogenous dissemination, and tissue necrosis [58];
- Dysregulation of ACE2 by SARS-CoV-2. By being present in many organs and tissues, the alteration of the receptor ACE2 expression causes a suitable environment for the development of mucormycosis. During COVID-19, a downregulation of ACE2 has been observed in the lungs [161], which causes inflammation, leukocytes exudation, and altered pulmonary function and therefore poor oxygenation [144]. Additionally, the effect of the virus on ACE2 in the pancreatic beta cells causes hyperglycemia, while dysregulation of the receptor in the vascular endothelium causes endothelial damage and vascular thrombosis, leading to vascular endothelial injury and venous stasis, causing an increase in serum iron due to hemolysis [144,145];
- Prolonged hospital stays and mechanical ventilation in severe COVID-19 cases expose the patient to fungal coinfections [15,103].

In addition, the use of industrial oxygen in COVID-19 patients due to a shortage of medical oxygen and the re-use of oxygen masks is suggested to be related to the development of CAM, especially in third-world countries. Unlike medical oxygen, industrial oxygen is not purified nor stored in disinfected cylinders [141,162].

### 3.1. Clinical Manifestations

In patients with the rhino-orbital/cerebral form, the most commonly observed symptoms are orbital/facial pain and edema, followed by loss of vision, nasal blockage, and ptosis [163]. These patients may also present fever, headache, ear pain, nasal blockage and discharge, eyelid edema, conjunctival swelling, vision loss, proptosis, extraocular muscle and cranial nerve palsy, diplopia, periorbital pain, orbital/facial discoloration, black nasal crusts, periocular hypoesthesia, palatal ulcer, toothache and lose teeth, epistaxis, and facial deviation [163,164,165,166]. Computed tomography (CT) or magnetic resonance imaging (MRI) can show sinusitis, erosion of the nasal septum, oroantral fistula, hard palate, air in bony sinus structures, focal mucosal non-enhancement, panophthalmitis, orbital infiltration, skull base involvement, cerebral sinus thrombosis and vasculitis, border zone infarcts, and meningeal enhancement [166,167].

In patients with pulmonary involvement, the most common symptoms are fever, cough, dyspnea, and hypoxia, and radiographic findings might include consolidations, cavitary lung lesions, and bronchopleural fistula formations with empyema [5,16,168].

### 3.2. CAM Diagnosis and Treatment

Both the European Confederation of Medical Mycology (ECMM) and the Mycoses Study Group Education and Research Consortium have released a detailed guide to facilitate mucormycosis diagnosis and treatment [104], while the ECMM and the International Society for Human and Animal Mycology (ISHAM) have published a guideline to identify CAM and how to manage it [169]. 

The gold standard for mucormycosis diagnosis is culture and microbiological or histopathological examination of tissues from different lesions [104,145,170]. Direct microscopic inspection reveals wide, non-septate, ribbon-like hyaline hyphae [171]. Culture of clinical specimens is usually made on Sabouraud agar and potato dextrose agar at 37 °C since all *Mucorales* grow in these media in 2 to 7 days [172]. Histopathological examination of tissue samples reveals non-pigmented, thin-walled, ribbon-like hyphae, with right angle-branching [173]. However, sometimes and depending on the clinical form, the sample can be difficult to obtain, and its processing might damage the hyphae preventing its growth in culture [169,174]. In addition, morphological identification is challenging, since *Mucorales* can be misidentified as *Aspergillus* spp. [175]. In addition, blood cultures are usually negative even in disseminated cases [176]. 

For pulmonary mucormycosis, radiological features can be suggestive of the infection, but these are non-specific and can overlap with other fungal cases of pneumonia or COVID-19. A pulmonary CT scan can be used for the detection of the reverse halo, which is observed as ground-glass opacity surrounded by a ring of consolidation mainly in the peripheral locations of the lungs [104]. However, this feature can overlap with findings associated with SARS-CoV-2 infection, which results in missed or late diagnoses [177]. Besides, CAM can also be mistaken for other fungal infections, such as pulmonary aspergillosis, which is highly associated with COVID-19 acute respiratory distress syndrome. Cavitary lung lesions can be a little more specific when trying to distinguish between COVID-19 and fungal infection, but both mucormycosis and aspergillosis can present these lesions, making it hard to identify the etiological agent of the mycosis [178]. In rhino-orbital/cerebral mucormycosis, radiological features are more specific than those for pulmonary infection. CT and MRI of the brain and paranasal sinus can be used to observe mucosal thickening and sinuses opacification, edema, inflammation, or brain infarction [169].

Other techniques, such as conventional and quantitative PCR (polymerase chain reaction) targeting the internal transcribed spacer (ITS) [179], semi-nested PCR targeting the 18S and ITS [180], PCR and RFLP (restriction fragment length polymorphism) analyses of the 18S [181], and DNA sequencing of the *FTRI* [182,183,184,185,186,187,188,189], have been used, but despite being highly specific, these might vary in sensitivity, are not widely available, or are extremely expensive [104]. PCR techniques alone can identify only certain species, including *Mucor*, *Rhizopus*, *Lichtheimia*, and *Rhizormucor* [33,184,185,190,191], but are not fully standardized [104]. PCR combined with matrix-assisted laser desorption/ionization-time of flight (MALDI-TOF) can also be used for species-level identification with higher accuracy [104,192]. In addition, MALDI-TOF mass spectrometry has been used to identify species-specific fungal peptides from fungal isolates or direct clinical specimens in a fast and sensible way [193,194,195]. Finally, a breath-based biosensor has been also proposed, where the volatile sesquiterpene metabolite profiles from murine models is analyzed by thermal desorption gas chromatography/tandem mass spectrometry (GC-MS/MS), obtaining different profiles for each *Mucorales* species, which can also be distinguished from aspergillosis [196]. However, these diagnosis techniques are expensive and need specialized equipment, hindering their daily use.

Currently, there are no serological diagnostic methods for mucormycosis [197], but some serological assays have been tested, including immunohistochemistry [187,188,189], ELISA (enzyme-linked immunosorbent assays), immunoblots, immunodiffusion, and 1,3-β-D-glucan assay, with different levels of success [198,199]. In addition, a lateral flow immunoassay (LIFA) was developed using a monoclonal antibody against the cell wall fucomannan of *Mucorales*, proving to be highly sensitive for the diagnosis of invasive murine mucormycosis [199].

Some carbon assimilation commercial kits are available for the identification of the *Mucorales* species, such as the ID32C and API 50CH kits, that successfully identify species such as *Lichtheimia corymbifera*, *Lichtheimia ramosa,* and *Rhizomucor pusillus* [194,200]. However, these kits are not capable of identifying all of the many *Mucorales* species that cause mucormycosis and have shown high variability in the assimilation patterns among *R. oryzae* isolates [200]. Furthermore, antifungal susceptibility testing can be used for the identification of the etiological agent, including methods such as broth microdilution, disk diffusion, and agar screening, and the use of the commercial kits YeastOne and VITEK2, but these methodologies are available only for a few antifungals, and their results are often hard to interpret [201].

Therefore, diagnosis of CAM mostly relies on conventional culture and histopathological demonstration, which is time-consuming and with low sensitivity, delaying diagnosis and treatment [177].

A metanalysis of published cases of CAM suggests that this infection is associated with high morbidity and mortality, mainly due to late diagnosis [177]. This study found that mucormycosis was diagnosed at a median of 10 days after COVID-19 diagnosis; for hospitalized patients that did not have any sign of mucormycosis on the day of admission, diagnosis took about 14.5 days after admission; in patients with uncontrolled diabetes, CAM was diagnosed in a median of 3.5 days after COVID-19 diagnosis, while in patients with controlled diabetes, diagnosis took 20 days; patients admitted to the ICU for COVID-19 advanced critical care were diagnosed at a median of 8 days after admission to the ICU. Diagnosis in these patients was made by histology or culture, and in some cases, samples were obtained post mortem [177]. All-cause mortality was reported in 49% of patients: for rhino-orbital/cerebral mucormycosis, mortality was reported in 37% of patients; for pulmonary, gastrointestinal, or disseminated mucormycosis, mortality was reported in 81% of patients. Among survivors with rhino-orbital/cerebral disease, loss of vision was reported in 46% of patients [177]. These data demonstrate that even in hospitalized patients, mucormycosis diagnosis takes too long, delaying treatment and increasing the patient’s mortality and risk of developing long-term side effects.

The therapy approach for the management of CAM is similar to that of mucormycosis, and the early diagnosis is essential for a good outcome [104,169]. The first step in treating CAM is to control or eliminate underlying predisposing factors, such as diabetes, acidosis, and corticosteroid use [104,169]. After this, the first recommended therapy is surgical resection and debridement of the affected tissues [104,169,202], followed by antifungal therapy for at least 6 weeks [104,141,169]. Amphotericin B, preferably its liposomal form, is the first-line therapy. In cases where amphotericin B cannot be used, posaconazole and isavuconazole can be used as salvage treatments [169]. Due to the current mucormycosis epidemic in India, a shortage of these antifungals has created the need to use other drugs. Although itraconazole is not recommended for the management of mucormycosis, it can be used as intravenous therapy [169].

Due to the limitations of antifungal drugs, which include side effects and optimal dosage, nanosystems have been used for the delivery of amphotericin B via oral, topical or pulmonary routes with promising results [203]. In these systems, the toxicity of the drugs has been reduced, and the route of administration has been optimized. In addition, the use of silver and zirconium oxide nanoparticles and nano-emulsion NB-201 themselves exhibit antifungal properties against *Mucorales* [204].

When CAM is being treated during active SARS-CoV-2 infection, potential drug-drug interactions of antifungals, antivirals, and immune therapies need to be addressed. For example, the administration of amphotericin B with steroids can cause hypokalemia in the patient, complicating the disease [141].

As new treatments are being tested for CAM, statin therapy has been suggested [205]. Statins have been shown to induce cytoprotective GRP-78 expression, decrease infection risk, and enhance the anti-CAM drug levels in plasma, as has been observed in in vitro studies with lovastatin, which causes apoptosis of *Mucor racemosus*, and gluvastatin and rosuvastatin, which have fungicidal effects against *Rhizomucor* and *Rhizopus* spp. [206]. Moreover, the combination of amphotericin B and atorvastatin/lovastatin showed a better effect against *R. arrhizus* than amphotericin B monotherapy [206].

In addition, anti-CotH3 and CotH7 antibodies have been found to protect mice with neutropenia and diabetes ketoacidosis against mucormycosis [207], while anti-integrin β1 antibodies inhibit *R. delemar* damage of alveolar epithelial cells in mice with diabetes ketoacidosis infected with pulmonary mucormycosis [208].

The use of hyperbaric oxygen has been proven beneficial as a possible adjunctive treatment, especially in patients with diabetes [209], due to the fungicidal activity that high concentrations of oxygen have against *Mucorales* in vitro [210].

Probiotics, due to their anti-inflammatory activity and antifungal and anti-mycotoxigenic activities [211,212,213,214,215], are also being proposed as a treatment for CAM because although their efficiency has not been proven for *Mucorales*, it has been demonstrated in candidiasis and other fungal infections [216,217,218,219] and for COVID-19 with good results [220,221,222,223,224].

A treatment being proposed for COVID-19 and some fungal infections that is gaining popularity are retinoids or vitamin A. Retinoic acid, the retinol biologically active metabolite, regulates many mechanisms in the host, including inflammatory effects, innate and adaptative immune responses, cell proliferation and differentiation, antiviral effects, and angiogenesis [225,226,227]. Retinoids increase the differentiation of respiratory epithelial cells, which might reduce the pathogen shedding period of infected cells and accelerate the repair processes of epithelial damaged by infection or inflammatory response [228]. However, retinoids also cause inflammation, dryness, and increased fragility of the mucosal epithelia, probably facilitating the penetration and colonization of the pathogen in the host [229,230]. Vitamin A is also related to the stimulation of NK, DCs, and innate lymphoid cells, promotion of the secretory IgA response, IL2 receptor transcription, and lymphocyte development and maturation, enhancing the immune response [225,227]. Finally, retinoids have inhibitory effects on diseases with aberrant angiogenesis [231], such as that observed during COVID-19. It has been observed that retinoids reduce ACE2 expression, reducing the risk of infection, and it is suggested they might inhibit the protease activity of SARS-CoV-2, decreasing viral proliferation [232]. Mega doses of vitamin A are proposed as an affordable adjunct therapy for COVID-19 with minimal side effects [233]. Although there are no reports of retinoids activity against *Mucorales*, it has been proven they have in vitro and in vivo antimycotic activity against *Candida albicans*, *Candida glabrata, Trichophyton* spp. [234], and *Aspergillus niger* [235], for which they are thought to be a possible treatment candidate for mucormycosis and thus also for CAM.

Drugs in the preclinical investigation, such as VT-1161, APX001A, and hemofungin, have been suggested as potential anti-*Mucorales* agents [236]. VT-1161 is a fungal CYP51 inhibitor gene responsible for voriconazole and fluconazole resistance in *R. oryzae*, which has shown a strong therapeutic action against *Rhizopus* spp., as observed by the increased survival rates of neutropenic mice with mucormycosis [237,238]; APX001A or fosmanogepix inhibits an important step of the glycosylphosphatidylinositol inositol post-translational pathway of surface proteins, which protects from *Rhizopus* infection in immunosuppressed mice [239]; hemofungin inhibits the in vitro growth of several *Mucorales*, including *Rhizopus* spp., and inhibits the final step of heme biosynthesis [240].

### 3.3. CAM Prevention and Outcome

The occurrence of CAM can be reduced up to a certain point, following several steps as suggested by [169]:

- During COVID-19 management, hyperglycemia should be strictly controlled;
- Corticosteroid therapy should only be used in patients with severe SARS-CoV-2 infection, for short periods and at low doses, just like other drugs that target the immune response, such as tocilizumab [210];
- The patient’s exposition to *Mucorales* should be limited as much as possible by using face masks and avoiding being outdoors;
- Mass vaccination against COVID-19 can also help to reduce the chance to develop severe secondary complications [210].

Even with treatment, CAM morbidity and mortality is high, being about 40–50% in prevalent regions and 50–100% in non-prevalent regions [210,241], but the prognosis might vary depending on the site of involvement, with poorer prognosis in cases of rhino-orbital/cerebral form [5]. Additionally, early diagnosis is a key determinant of the prognosis, and due to the rapid dissemination of the infection, delayed treatment initiation can result in a twofold mortality increase (up to 80%) [210].

## 4. Concluding Remarks

The recent rise of the COVID-19 pandemic has highlighted the importance of mucormycosis worldwide. The lack of knowledge about mucormycosis and its etiological agents has complicated CAM diagnosis, treatment, and prevention and therefore has worsened the clinical management of COVID-19 patients, affecting health and the economy globally. Many efforts have been made to understand SARS-CoV-2 and to prevent and treat COVID-19, but the study of fungal coinfections, such as mucormycosis, is a priority for both basic and clinical research.

To improve the current global crisis, the study of opportunistic pathogens such as *Mucorales* needs to be addressed urgently. For example, neither molecular nor serological diagnosis methods are commercially available for the daily and rapid identification of this mycosis, which hinders diagnosis and delays treatment. Future efforts should be made in finding fungal antigenic molecules and host antibodies generated against them, developing fast and accessible molecular and serological diagnostic methods, and also in finding molecular markers for the identification of the mucormycosis etiological agents. The different antifungal resistance profiles of *Mucorales* species highlight the importance of fungal identification at the species level. Moreover, research in new antifungal therapies is needed to improve the infection outcome and decrease the mortality rate. As mentioned before, the typical antifungal drugs currently used have different responses depending on the etiological agent and are usually not very effective for severe cases if administrated out of time. Moreover, since they have to be used in high doses and for long periods, they can be toxic, permanently damage organs, and can generate resistance. In addition, the shortage of these drugs in regions where mucormycosis is endemic is of concern since it limits the treatment of many patients with severe forms of mycosis. Therefore, due to the lack of rapid and accessible diagnosis, treatment for mucormycosis can be challenging. One alternative to overcome these hurdles is to focus future research efforts on drug repurposing, looking for already known drugs with undiscovered antifungal activity.

There is not much that can be done to prevent CAM; simply, certain measures can be taken, such as avoiding mucormycosis risk factors, which can be complicated for patients with underlying diseases and in regions with poor healthcare systems. Therefore, we conclude that research efforts in mucormycosis are as important as those in COVID-19 to fight the current pandemics and associated infections.

## Data Availability

Not applicable.
